# Supplementary material for: The rosetteless gene controls development in the choanoflagellate S. rosetta
Source: eLife. 2014 Oct 9;3:e04070. doi: 10.7554/eLife.04070 (PMC4381721; doi:10.7554/eLife.04070)
Supplement: Supplementary file 1. — Primers used for genotyping and assessing splicing. DOI: http://dx.doi.org/10.7554/eLife.04070.020 [file elife04070s002.doc]

Supplementary file 1.

**Primers used for genotyping and assessing splicing**. Primers used to genotype microsatellites by fragment analysis (MF), genotype microsatellites by 2% agarose gel (MG), verify and genotype SNPs using Sanger sequencing (SNP), assess the splicing of EGD82922 from cDNA (SPL), clone the epitope for the anti-Rtls antibody (E), or sequence the coding region of EGD82922 from gDNA (C). One primer was directly fluorescently labeled with 6-carboxyfluorescein (6FAM).

| **Primer name** | **Genomic position*** | **Primer use** | **Primer sequence** |
| --- | --- | --- | --- |
| gt_indel_7 | sc2: 1,730,188 | MF | TGTAAAACGACGGCCAGTCACAACAGAGAAGCCGGATG |
|  |  |  | GCACATGAGCGCTTAGAATTC |
| gt_indel_2 | sc7: 855,120 | MF | TGTAAAACGACGGCCAGTGCCTTATCAACTCCCTGTGC |
|  |  |  | GCGTGATTGCCTTCGTTTCA |
| gt_indel_9 | sc8: 1,073,581 | MF | 6FAM-ATGGGCGCCTTGAACAAAC |
|  |  |  | TGCTCCAGATGTCAGCTTCA |
| gt2 | sc8: 427,804 | SNP | GGACACGCGAGACATAGACA |
|  |  |  | TTTCATTTGGGATACGCACA |
| gt3 | sc22: 1,229,741 | SNP | GAAATTCACGGTGACGGACT |
|  |  |  | ACGCAATCGGTAAAGGTCAC |
| gt15 | sc1: 401,602 | SNP | CAATTGCACCAAGCAAGAGA |
|  |  |  | TGCATGTCTCTCACGAGTCC |
| gt18 | sc5: 1,770,007 | SNP | GCTATGGTGGCGAGTACGTG |
|  |  |  | CTGATAGCACTTGCGGCATA |
| gt23 | sc29: 385,389 | SNP | GGGCATCCAACACATACACA |
|  |  |  | TCGTCCGTTCAAACTCTTCC |
| indel1 | sc22: 391,927 | MG | GGAACAGGGAGCCACAACTA |
|  |  |  | ACGCTCGCCTACTACGACTC |
| Rtls_2L | Spans exons 5 and 6 of *EGD82922* | SPL | CCGCATTTGAGGCCGAAT |
| Rtls_5L | Spans exons 7 and 8 of *EGD82922* | SPL | GACTGAATTCCCCGCCATC |
| Rtls_1R | Spans exons 10 and 11 of *EGD82922* | SPL & C | CAGCATCAATCACGCACTGA |
| Rtls_4L | sc8: 430573 | C | GCTATGTGGAGTTCTCCGATG |
| Rtls_2R | sc8: 426601 | C | TGAACGATGCACTGGGTTG |
| Rtls_L1 | sc8: 431369 | C | CTGCGCCCGTTGATGTTG |
| Rtls_L3 | sc8: 428490 | C | GTGGCCCTCGCTTGTGGT |
| Rtls_L4 | sc8: 431240 | C | CACACACAGGTCGTTTGCTT |
| Rtls_L5 | sc8: 429836 | C | CGACAACGACGAGAGAGACA |
| Rtls_L6 | sc8: 429763 | C | CAATCCAGAAGTGCCAGGTT |
| Rtls_R1 | sc8: 428389 | C | AAAACGCGTTCCTCCACTGC |
| Rtls_R2 | sc8: 425565 | C | GTGGTGGAAGGTGCAACAGG |
| Rtls_R3 | sc8: 429763 | C | AACCTGGCACTTCTGGATTG |
| Rtls_R4 | sc8: 428351 | C | GATTGCAGTGAGGGGTTCAC |
| Rtls_R6 | sc8: 427237 | C | CGTGGACGATGTTGTCCTAA |
| Rtls_epit_L1^†^ | sc8: 429199 | E | ATTGGATCCTCCTCGACGCCACAACAGT |
| Rtls_epit_R1^†^ | sc8: 428696 | E | CGGAATTCTTAGGTGGCCTGAATCTGGTCAAG |

* Indicated either as supercontig (sc): nucleotide position or as sequence bridging two exons in *EGD82922* (*rtls*).

† These primers include the restriction enzyme sequences used to clone the Rtls epitope into the expression vector
